# 离子色谱法同时测定脱硫液中8种有机胺类物质

**DOI:** 10.3724/SP.J.1123.2023.09024

**Published:** 2024-05-08

**Authors:** Lihong GAO, Zhenbang JIANG, Hongguo ZHENG, Wenhui LI

**Affiliations:** 1.北京科技大学化学与生物工程学院, 北京 100083; 1. School of Chemistry and Biological Engineering, University of Science and Technology Beijing, Beijing 100083,China; 2.赛默飞世尔科技(中国)有限公司, 北京 100102; 2. Thermo Fisher Scientific Inc., Beijing 100102, China

**Keywords:** 离子色谱, 有机胺类物质, 脱硫液, ion chromatography (IC), organic amines, desulfurization solution

## Abstract

有机胺脱硫工艺因高效、经济等优点,应用越来越广泛。不同配比的有机胺对二氧化硫脱除效果不同。因此,对脱硫液中的不同有机胺成分进行准确测定具有重要意义。离子色谱法检测有机胺不需要衍生步骤,前处理简单,并且可同时测定多种有机胺。本研究采用离子色谱法同时测定乙醇胺(MEA)、二乙基乙醇胺(DEEA)、*N*-甲基二乙醇胺(MDEA)、2-氨基-2-甲基-1-丙醇(AMP)、羟乙基乙二胺(AEEA)、哌嗪(PZ)、*N*-羟乙基哌嗪(HEPZ)、二乙烯三胺(DETA)等8种有机胺。实验对比不同型号色谱柱、淋洗液和柱温条件对8种有机胺的分离效果,最终采用IonPac CS17色谱柱,柱温35 ℃,甲基磺酸(MSA)水溶液梯度淋洗,抑制电导法进行测定。脱硫液样品采用超纯水稀释,过0.22 μm尼龙微孔滤膜和OnGuard Ⅱ RP柱后进样分析,样品前处理简便。8种有机胺在一定范围内具有良好的线性关系,判定系数*R*^2^≥0.9980。以信噪比(*S/N*)=3时对应的有机胺质量浓度为检出限(LOD),以*S/N*=10时对应的有机胺质量浓度为定量限(LOQ)。在进样量1.0 μL时,LOD即可达到0.02~0.08 mg/L, LOQ为0.07~0.27 mg/L。实际样品的加标回收率为93.0%~111%,相对标准偏差(RSD, *n*=5)为0.31%~1.2%,说明方法具有良好的准确性和精密度,适用于脱硫液中多种有机胺的测定。

## Abstract (en)

The applications of organic-amine desulfurization have steadily increased owing to its high efficiency, low cost, and low energy consumption. Different proportions of organic amines exert different effects on sulfur dioxide removal. Therefore, the accurate determination of different organic amines in the desulfurization solution is of great importance. The ion-chromatographic method for the detection of organic amines does not require a derivatization step, has simple pretreatment procedures, and allows for the simultaneous determination of many types of organic amines. In this study, a method based on ion chromatography was developed for the simultaneous determination of ethanolamine (MEA), diethylethanolamine (DEEA), *n*-methyldiethanolamine (MDEA), 2-amino-2-methyl-1-propanol (AMP), hydroxyethylethylenediamine (AEEA), piperazine (PZ), *n*-hydroxyethylpiperazine (HEPZ), and diethylenetriamine (DETA). The separation efficiency of the eight organic amines in different types of columns, leaching solutions, and column temperatures were compared. The determination was performed using an IonPac CS17 column with column temperature of 35 ℃ and gradient leaching with methyl sulfonic acid (MSA) solution via the inhibition conductance method. Samples of the desulfurization solution were analyzed using ultrapure water filtered through a 0.22 μm nylon microporous filter membrane and an OnGuard Ⅱ RP column; thus, the pretreatment steps are simple. The eight organic amines showed a good linear relationship within a certain concentration range, and the coefficient of determinations (*R*^2^) were greater than 0.998. The limits of detection (LODs) and quantification (LOQs) were determined from the mass concentrations of the organic amines corresponding to signal-to-noise ratios (*S/N*) of 3 and 10, respectively. LODs of 0.02-0.08 mg/L and LOQs of 0.07-0.27 mg/L were determined from a 1.0 μL sample injection. The actual recoveries ranged from 93.0% to 111%, and the relative standard deviations (RSDs, *n*=5) ranged from 0.31% to 1.2%. The results indicated that the proposed method has good accuracy and precision; thus, it is suitable for the determination of various organic amines in desulfurization solution.

化石燃料燃烧产生的烟气中含有大量二氧化硫(SO_2_)气体,这些气体会造成环境空气污染,并且与人体的呼吸道疾病息息相关。烟气脱硫技术至关重要,有机胺脱硫工艺因高效、经济等优点,应用越来越广泛^[1]^。乙醇胺(MEA)和二乙醇胺等是早期常用的醇胺类脱硫剂。近年来,哌嗪类有机胺、多元胺、复合胺脱硫技术不断涌现,不同配比下的有机胺对SO_2_脱除效果不同。因此,对脱硫液中不同有机胺成分进行准确测定具有重要意义。

有机胺的常用检测方法包括液相色谱法(LC)^[2⇓-4]^、气相色谱法(GC)^[5⇓-7]^、离子色谱法(IC)^[8⇓⇓⇓⇓⇓-14]^、液相色谱-质谱法(LC-MS)^[15⇓-17]^、气相色谱-质谱法(GC-MS)^[18⇓-20]^等。其中,对于醇胺类有机胺,液相色谱法需要柱前或柱后衍生后检测,衍生步骤耗时耗力,同时可能引入其他污染;顶空气相色谱法可直接对挥发性有机胺进行测定,但水溶液中的有机胺通常难挥发不易萃取。刘凤娴等^[19]^采用GC-MS对大气颗粒物中有机胺类物质进行测定,在测定前仍然需要对样品进行衍生化处理。LC-MS可直接对有机胺类物质进行测定,唐泽坤等^[16]^采用亲水相互作用色谱-静电场轨道阱高分辨质谱法测定CO_2_捕集过程中9种有机胺类化合物,样品无需衍生,方法简便,但仪器成本较高。离子色谱法利用有机胺的阳离子性质进行分离,抑制电导法进行测定,样品无需衍生,前处理简单,一次进样可同时分析多种有机胺,并且仪器成本较低,同时无需使用甲醇或乙腈等有机试剂,对环境和操作人员更加友好。

本研究采用离子色谱电导法测定乙醇胺、二乙基乙醇胺(DEEA)、*N*-甲基二乙醇胺(MDEA)、2-氨基-2-甲基-1-丙醇(AMP)、羟乙基乙二胺(AEEA)、哌嗪(PZ)、*N*-羟乙基哌嗪(HEPZ)、二乙烯三胺(DETA)等8种有机胺类物质。该方法经济实用,将其应用于脱硫液中有机胺的测定,可为有机胺脱硫技术的研究提供技术支持。

## 1 实验部分

### 1.1 仪器、试剂与材料

Dionex ICS-6000高压离子色谱仪(配有自动电解淋洗液发生器)和OnGuard Ⅱ RP柱(美国赛默飞世尔科技(中国)有限公司), 0.22 μm尼龙微孔滤膜(上海安谱实验科技股份有限公司)。

MEA(纯度99.5%)、AMP(纯度98%)、AEEA(纯度99%)、PZ(纯度99%)、HEPZ(纯度98%)、DETA(纯度99%)均购自百灵威科技有限公司;DEEA(纯度99%)和MDEA(纯度99%)均购自上海阿拉丁生化科技股份有限公司;18.2 MΩ·cm超纯水由Barnstead GenPure Pro超纯水机(美国赛默飞世尔科技(中国)有限公司)制备。

### 1.2 前处理条件

本实验所用的4份脱硫液来自某研究院某项目现场样品,其中1~3号样品为高含量有机胺平行样,4号为低含量有机胺样品。由于不同样品间有机胺含量差异较大,因此1~3号样品采用超纯水稀释1000倍,4号样品采用超纯水稀释100倍,然后过0.22 μm尼龙微孔滤膜和OnGuard Ⅱ RP柱后进样分析。

OnGuard Ⅱ RP柱使用前依次用5 mL甲醇和10 mL超纯水活化,放置15 min后使用,样品过柱时弃去前3 mL流出液。

### 1.3 色谱条件

分析柱:IonPac CS17(250 mm×4 mm);保护柱:IonPac CG17(50 mm×4 mm);柱温:35 ℃;淋洗液:甲基磺酸(MSA,淋洗液发生器自动电解产生);梯度洗脱程序:0~19 min, 1 mmol/L; 19~34 min, 1~17 mmol/L; 34~41 min, 17 mmol/L; 41~41.1 min, 17~1 mmol/L; 41.1~45 min, 1 mmol/L。流速:1.0 mL/min;定量环:1.0 μL;抑制器:CDRS 600 (4 mm),自循环电解抑制器,抑制电流50 mA;电导检测器,电导池温度35 ℃。

## 2 结果与讨论

### 2.1 色谱柱的选择

目前常用的阳离子色谱柱中,IonPac CS17和CS19是专门为测定有机胺类物质开发的色谱柱。本研究比较了这2种色谱柱对8种有机胺的分离效果,如图1所示,CS19色谱柱对DETA和HEPZ的分离效果不佳,分离度小于1.5,并且在CS19柱上,HEPZ出现峰分叉的现象;而CS17色谱柱对8种有机胺均有良好的分离效果,8种有机胺分离度均大于2,因此本研究最终选用CS17色谱柱。

**图1:**
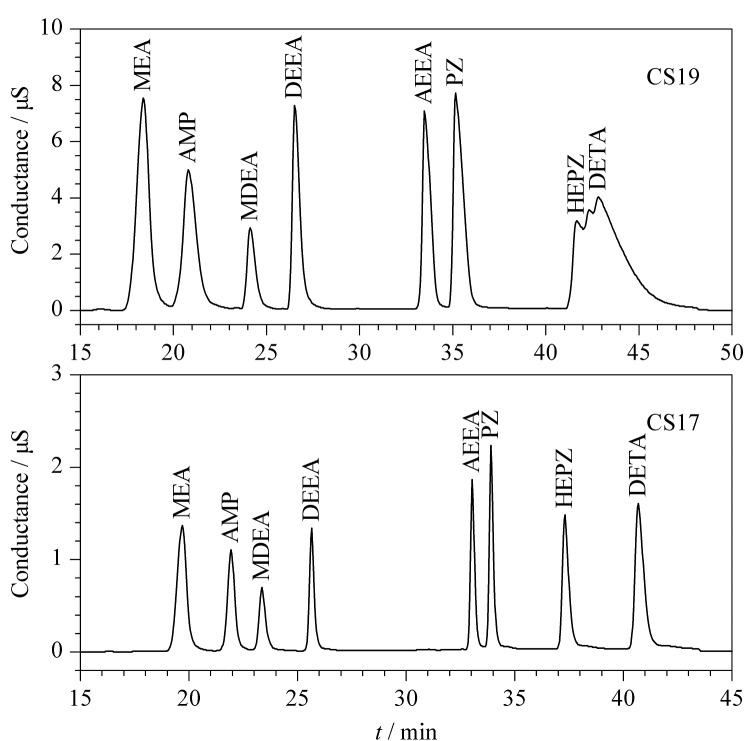
CS19和CS17色谱柱分离8种有机胺的色谱图

### 2.2 柱温的选择

色谱柱温度也是影响分离和保留的重要因素。选用CS17色谱柱,分别比较25、30、35 ℃柱温条件下8种有机胺的分离情况,如图2所示。结果显示,柱温越高,MEA和AMP的分离度越大。此外,随着柱温的升高,各组分峰的保留时间都在减小,即柱温越高,分析时间越短。因此本研究最终选择35 ℃作为分析时的柱温。

**图2:**
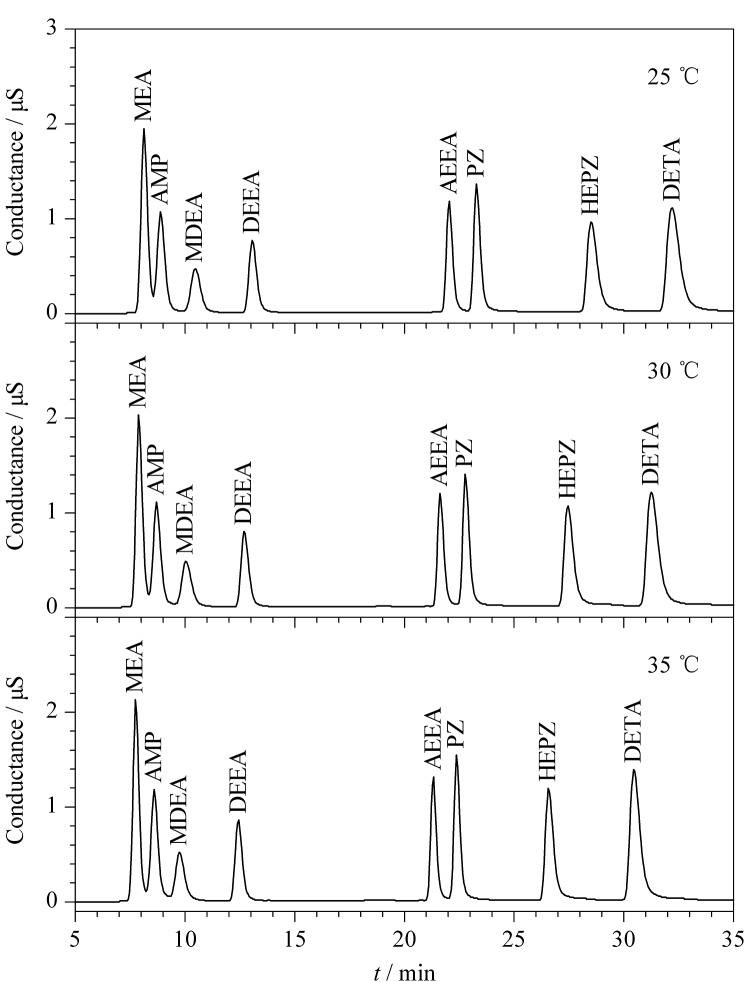
不同柱温条件下8种有机胺的色谱图

### 2.3 淋洗液洗脱程序的优化

除色谱柱外,淋洗液对目标物的分离和保留具有重要影响。MSA水溶液是阳离子分析时最常用的淋洗液。本研究中的8种有机胺,MEA和AMP保留较弱,需要低浓度MSA洗脱,而HEPZ和DETA保留较强,需要高浓度MSA洗脱。比较了不同梯度洗脱程序下8种有机胺的分离效果。如图3所示,采用初始浓度3 mmol/L的MSA洗脱时,MEA、AMP和MDEA不能完全分离,因此降低MSA初始浓度为2 mmol/L,同时延长初始浓度保持时间,MEA、AMP和MDEA的分离效果明显改善,继续降低MSA初始浓度至1 mmol/L, MEA、AMP和MDEA可达到完全分离。MSA浓度梯度升高至17 mmol/L并保持一定时间,可将其他有机胺完全洗脱和分离。最终优化的MSA梯度洗脱程序如1.3节所示。

**图3:**
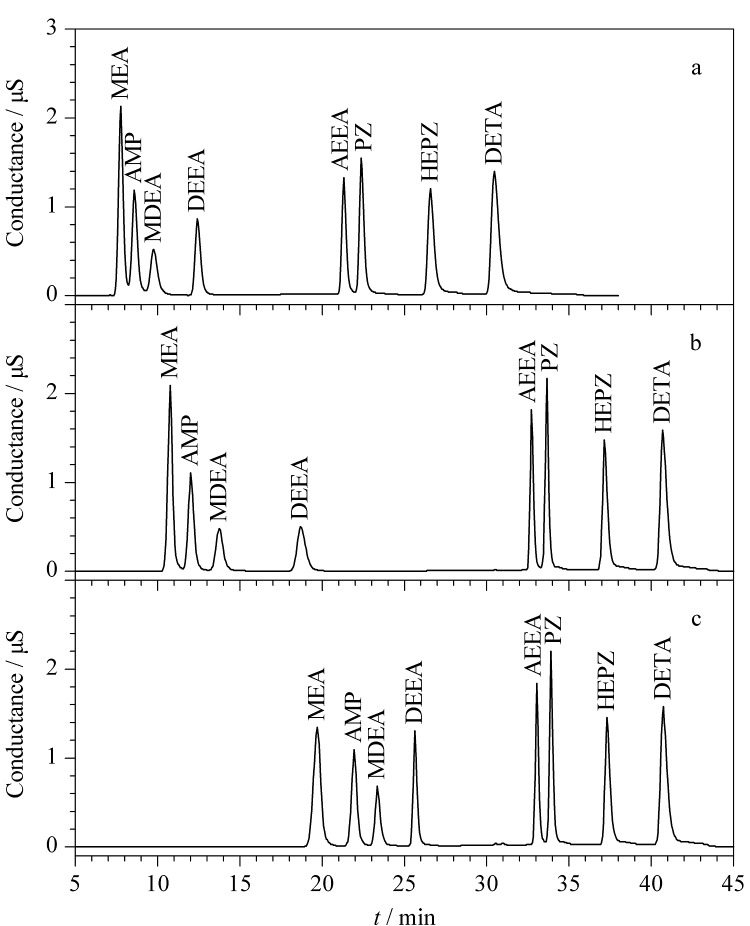
不同淋洗液梯度洗脱程序下8种有机胺的色谱图

### 2.4 线性范围、检出限和定量限

配制系列浓度混合标准溶液,在选定的色谱条件下进样分析,以各离子的色谱峰面积(*y*)对质量浓度(*x*, mg/L)绘制标准曲线。从结果可以看出,8种有机胺在10~200 mg/L范围内具有良好的线性关系,判定系数*R*^2^≥0.9980。以信噪比(*S/N*)=3时对应的有机胺质量浓度为检出限(LOD),以*S/N*=10时对应的有机胺质量浓度为定量限(LOQ)。

实验结果表明,在进样量1 μL时,检出限即可达到0.02~0.08 mg/L,定量限在0.07~0.27 mg/L范围内,数据见表1。

**表1:** 8种有机胺的线性范围、判定系数、检出限和定量限

Compound	Linear range/(mg/L)	R^2^	Linear equation	LOD/(mg/L)	LOQ/(mg/L)
MEA	10-200	0.9993	y=0.0071x-0.0011	0.05	0.17
AMP	10-200	0.9999	y=0.0044x-0.0137	0.08	0.27
MDEA	10-200	0.9994	y=0.0023x+0.0065	0.05	0.17
DEEA	10-200	0.9999	y=0.0036x-0.0060	0.04	0.14
AEEA	10-200	0.9980	y=0.0036x+0.0164	0.02	0.07
PZ	10-200	0.9990	y=0.0046x+0.0102	0.02	0.07
HEPZ	10-200	0.9992	y=0.0046x+0.0357	0.03	0.10
DETA	10-200	0.9988	y=0.0068x+0.0512	0.04	0.14

*y*: peak area; *x*: mass concentration, mg/L.

### 2.5 回收率与精密度

选取1份本底值较低的脱硫液样品,在其中加入低、中、高3个水平的8种有机胺标准溶液,每个水平做5个平行,测定8种有机胺的回收率和相对标准偏差(RSD),数据见表2。结果显示,8种有机胺的回收率为93.0%~111%, RSD(*n*=5)为0.31%~1.2%,说明方法具有良好的准确性和精密度。

**表2:** 8种有机胺在脱硫液中的加标回收率(*n*=5)

Compound	Background/ (g/L)	Added/ (g/L)	Found/ (g/L)	Recovery/ %	RSD/ %	Compound	Background/ (g/L)	Added/ (g/L)	Found/ (g/L)	Recovery/ %	RSD/ %
MEA	8.45	5.0	13.4	99.0	0.51	AEEA	nd	1.0	1.10	110	0.87
		7.5	15.7	96.7	0.97			5.0	5.27	105	0.47
		16	24.0	97.2	0.85			20	20.5	103	0.88
AMP	nd	1.0	0.97	97.0	0.93	PZ	nd	1.0	0.98	98.0	0.93
		5.0	5.29	105	0.46			5.0	5.23	104	0.43
		20	19.2	96.0	1.2			20	19.8	99.0	0.98
MDEA	10.1	5.0	15.0	98.0	0.40	HEPZ	6.10	2.5	8.68	103	0.31
		10	19.6	95.0	0.78			5.0	11.2	102	0.34
		20	29.3	96.0	0.95			12	17.9	98.3	0.63
DEEA	nd	1.0	0.93	93.0	0.75	DETA	nd	1.0	1.07	107	0.84
		5.0	5.01	100	0.52			5.0	5.57	111	0.47
		20	19.6	98.0	1.0			20	20.3	102	0.68

nd: not detected.

### 2.6 实际样品分析

准确量取脱硫液样品,1~3号脱硫液用超纯水稀释1000倍,4号脱硫液用超纯水稀释100倍,过0.22 μm尼龙滤膜和OnGuard Ⅱ RP柱后,采用选定的色谱条件进行测定,外标法定量,测定结果如表3所示。在4份脱硫液样品中均检出MEA、MDEA和HEPZ,其他5种有机胺未检出。

**表3:** 脱硫液样品中有机胺的含量

No.	MEA	AMP	MDEA	DEEA	AEEA	PZ	HEPZ	DETA
1	101	<LOD	168	<LOD	<LOD	<LOD	56.5	<LOD
2	85.3	<LOD	140	<LOD	<LOD	<LOD	38.8	<LOD
3	82.0	<LOD	131	<LOD	<LOD	<LOD	33.2	<LOD
4	8.45	<LOD	10.1	<LOD	<LOD	<LOD	6.10	<LOD

## 3 结论

本研究建立了离子色谱法同时检测8种有机胺的方法。8种有机胺具有良好的分离度,样品经稀释和过柱后直接进样分析,无需衍生,操作简单,并且方法具有良好的准确性和精密度,适用于脱硫液中多种有机胺的测定,可应用于有机胺吸收剂SO_2_吸收工艺的研究中,为吸收剂中有机胺的成分和浓度分析提供检测技术。
